# Methylation index as means of quantification of the compliance of sedimentary mercury to be methylated

**DOI:** 10.1007/s10661-015-4716-y

**Published:** 2015-07-10

**Authors:** Jacek Bełdowski, Michał Miotk, Janusz Pempkowiak

**Affiliations:** Institute of Oceanology PAN, ul. Powstańców Warszawy 55, 81-712 Sopot, Poland

**Keywords:** Sediment characteristic, Methylmercury, Baltic Sea, Spitsbergen fjords, Sulfur-reducing bacteria, Redox conditions

## Abstract

Methylmercury (MeHg) is the most bioavailable and toxic mercury species in the marine environment. MeHg concentration levels, methylation rates leading to MeHg formation, and methylation index (MI) are all used to assess the compliance of mercury to be methylated in the marine sedimentary environment. This paper reports on the works conducted on the MI upgrade. This paper proposes a new formula for calculating MI. Apart from labile mercury(II) and organic matter, it includes redox potential and abundance of sulfur-reducing bacteria (SRB), both essential factors for MeHg generation. The obtained MI is validated against actual sedimentary MeHg concentrations proving the potential usefulness of MI as a factor characterizing status of sedimentary environment regarding possible occurrence of MeHg. Moreover, values of the methylation index in particular regions show that MI values correspond well to environmental conditions in those areas. The values calculated correlate well with MeHg concentrations; however, the correlation coefficients vary between different regions. This has been attributed to the lack of empirical coefficients. Thus, MI could be used as a characteristic of the sedimentary environment indicating the potential presence of MeHg. It could also be used in methylation rate modeling, provided that empirical constants are applied to improve model performance.

## Introduction

Methylmercury (MeHg) is the most bioavailable mercury species in the marine environment (Boening 2000). Due to its hydrophobic character, it undergoes biomagnification in the food chain and is capable of penetrating blood/brain and blood/placenta barriers (Buccolieri et al. 2006). Moreover, it is a neurotoxin, as it damages the nerve system and brain (Boening 2000). Therefore, environmental chemists have investigated the mechanism of MeHg generation and its occurrence in the environment (Hintelmann et al. 2000; Jackson 1998; Avramescu et al. 2011) including the marine sediments (Heyes et al. 2006; Monperrus et al. 2007) for several decades.

Mercury methylation in the marine environment may be attributed to both biotic and abiotic processes (Cossa et al. 2009). Biological methylation includes the transfer of methyl groups from methyl cobalamine by bacteria to ionic mercury(II). Sulfur-reducing bacteria (SRB) are suggested as the mediating organism (Figueiredo et al. 2011). The mechanism, though, is not fully understood, and the rate of mercury methylation is believed to depend on many environmental factors—including the availability of mobile mercury(II), the abundance and activity of microorganisms, sulfate concentration, and redox potential in sediments (Jackson 1998; Choi et al. 1994).

The factors listed above are of varied importance to methylation. Mobile mercury(II) presence in the sediments acts as a substratum for methylation; therefore, its abundance is clearly the most important factor controlling methylation rate. Mercury mobility in the sediments can be either deduced from total mercury concentrations and environmental factors such as the content of labile organic matter or obtained experimentally, i.e., by sequential extraction (Wallschlager et al. 1998; Bełdowski and Pempkowiak 2003). Total sulfur is believed to play a role in mercury availability for methylation and is also an indicator of the presence of sulfur-reducing bacteria (Schartup et al. 2014). The role of SRB in mercury methylation is crucial—experiments in microcosms have showed that a lack of SRB results in halving or even quartering methylation rates (Avramescu et al. 2011). Organic carbon and redox potential are both linked to SRB activity. SRB are obligate anaerobes that oxidize organic substances using sulfate as the terminal electron acceptor (Harmon et al. 2007). In many areas, including the Baltic Sea, organic carbon rich sediments are anoxic (Bełdowski and Pempkowiak 2003), causing these two factors to act jointly.

Abiotic methylation is possible whenever easily accessible methyl groups are available and has been observed both in sediments and in the water column (Cossa et al. 2009; Krishnamurthy 1992). When this occurs, methylation is induced by small organic molecules, such as dimethyl sulfide or methyl iodide or larger molecules, such as those of humic or/and fulvic acids. It is, however, generally accepted that microbial methylation is significantly faster (Choi et al. 1994).

Since methylmercury concentrations can display significant patchiness (Bełdowski et al. 2014; Pempkowiak et al. 1998), and measurements of this mercury species are lengthy and complicated, other approaches have been postulated. These are based on the quantification of mercury compliance to be methylated in situ. To this end, numerous approaches to estimate the methylation rate in the environment were undertaken (Avramescu et al. 2011). One of these was a methylation index—a sediment characteristic combining factors influencing mercury methylation—which was proposed for this purpose by us (Bełdowski et al. 2009). The ability of sediments to methylate mercury, whether expressed as methylation rates or a biogeochemical index may be more important than actual MeHg concentrations, since the latter represent only a momentary situation, while the former could be utilized to predict long-term environmental trends.

Both, direct analysis of MeHg in sediments (Pak and Bartha 1998) and analyses of operationally defined organic mercury (Boszke et al. 2007) have been used to assess the ability of sedimentary mercury to be methylated. The former approach is, by far, more popular although MeHg quantification is troublesome (Liang et al. 1994). Organic mercury, on the other hand, apart from methylmercury, comprises other species of mercury covalently bound to carbon and mercury(II) complexed by humic substances, and thus lacks the required specificity (Bełdowski et al. 2014).

Environmental concentrations of MeHg reflect net methylation rather than an actual MeHg synthesis rate resulting from both biotic and abiotic processes and represent a balance between methylation and demethylation (Avramescu et al. 2011). According to recent studies (Merritt and Amirbahman 2008), these two processes overlap spatially and/or kinetically, and their balance conditions the actual abundance of sedimentary MeHg. Therefore, kinetic models were suggested as a solution for net methylation rate determination. Multiple studies using radioactive and stable mercury isotopes were undertaken to assess both methylation and demethylation rates (Hintelmann et al. 2000; Hintelmann and Ogrinc 2003). Pseudo-first-order reaction is, most often, assumed, and the reaction constants K are used to characterize the processes.

In freshwater, the methylation constant (Km) varies from 0.01 to 0.06 day^−1^, while demethylation (Kd) is in the range from 0.07 to 15.84 day^−1^ (Avramescu et al. 2011 and references therein). In the marine environment, i.e., the Hudson River Estuary and Bay of Fundy such rates ranged from 1.05 × 10^−4^ to 1.11 × 10^−3^ (Heyes et al. 2006). Methylation rate is highly heterogeneous even at short distances as variability of up to 50 % were reported (Monperrus et al. 2007). Within the Thonde Estuary methylation rates (expressed as the methylated fraction (%) of total Hg day^−1^) ranged from 0.25 to 1.32—amounting to 12 nmol m^−2^ day^−1^ (Monperrus et al. 2007).

Another approach toward characterizing mercury methylation ability of sediments was proposed by us in 2009 (Bełdowski et al. 2009). The authors named it the methylation index (MI). The methylation index was defined as a product of the important sediment features that condition mercury methylation: concentrations of both mobile mercury and organic matter. The index was proven to be well correlated with mercury levels in fish; however, no relation to sedimentary MeHg was assessed. The “methylation index” approach as a feature characterizing sedimentary environment has then been further elaborated within this study, to include the activity of SRB, organic matter, and mobile mercury(II). The usefulness of the new MI has been tested in several marine sedimentary environments with differing concentrations of mobile mercury(II), organic matter, and SRB activity. This has resulted in the development of a formula on sediment methylation index that is well correlated to the concentration of sedimentary MeHg. MI can be used as a sediment characteristic providing information on the compliance of a given sedimentary environment for mercury methylation without actually analyzing this deadly mercury species.

This paper reports on the works conducted on the MI upgrade to include relevant environmental parameters for mercury methylation and to validate it against actual MeHg concentrations in diversified sedimentary environments. The investigated sediment samples were collected in both the contaminated Baltic Sea and in a reference area of the Svalbard Archipelago (European Arctic).

## Materials and methods

### Study areas

Sediments for this study were collected in two areas, differing in sediment composition and anthropogenic impact—the southern Baltic Sea and the Spitsbergen Fjords.

**Fig. 1:**
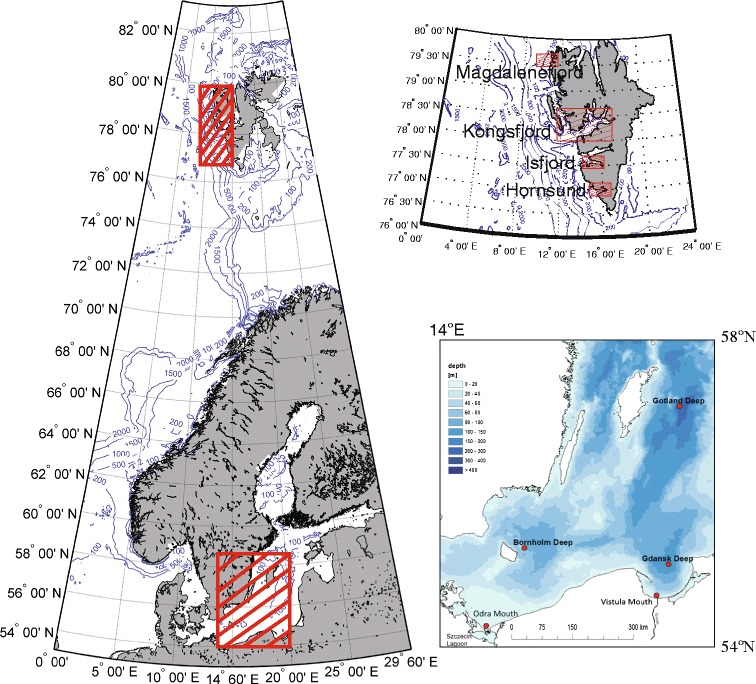
Study areas and distribution of sampling stations

The Baltic Sea is a semi-enclosed water body surrounded by highly industrialized countries. Geochemical cycles in the Baltic have been strongly influenced by human activities since the beginning of the twentieth century (Borg and Jonsson 1996; Pempkowiak 1991). For this study, both offshore sediment accumulation areas and coastal areas close to the river mouth were sampled. The former were characterized by organic carbon contents in the range from 3.4 to 6.2 % accompanied with reducing conditions, while the latter comprised organic matter in the range from 0.2 to 1.4 %, and oxic conditions. Detailed description of this study area may be found in (Bełdowski et al. 2014). Spitsbergen is Norway’s largest island. Over 50 % of the island is covered with glaciers and mountains made of sedimentary rocks with steep slopes and flat tops. Metamorphic and volcanic rocks are characteristic to the western shores of Spitsbergen where the sampled fjords are located. The shoreline of the island is deeply cut with numerous fjords. Four of the fjords were sampled for sedimentary mercury analysis. Spitsbergen is a remote area, where most of the mercury comes predominantly from rock weathering (Bełdowski et al. 2015). Detailed description of this study area may be found in Bełdowski et al. (2015). Location of sampling areas is shown in Fig. 1.

### Sample collection and analysis

Samples were collected by the r/v Oceania during 2010–2011 with a gravity corer. The top three centimeters of stratified sediments were sampled by cutting it away with a plastic spatula, mixing, transferring into polyethylene bags, and storing frozen (−20 °C) until analyses in laboratory.

All the samples were homogenized under a laminar flow hood, and aliquots were taken for further analyses. Those included organic carbon, redox potential, SRB abundance, total mercury, mobile mercury fractions, and methylmercury.

Organic carbon content in sediments was determined after the removal of carbonates (2 M HCl) using an Elemental Analyzer Flash EA 1112 Series combined with the Isotopic Ratio Mass Spectrometer IRMS Delta V Advantage (Thermo Electron Corp., Germany) and presented as percentage in the bulk of the dry sample. Quality control was carried out with standard materials supplied by the Thermo Electron Corp. The methodology used provided satisfactory accuracy (given as recovery) and precision—given as relative standard deviation (average recovery 99.1 ± 2.0 % RSD; *n* = 5).

Redox potential values were measured in subsamples of sediments, immediately after sediment sampling with an automated electrochemical meter produced by WTW (Germany). Redox electrodes were calibrated before each series of analyses with certified NIST buffers.

SRB were analyzed according to Martinez et al. (2004) and Richir et al. (2012). The water for analysis was collected directly above the sediment-water interface with a sterilized syringe, filtered through precleaned glass filters, and placed in test tubes—BART kit tests (HACH). Tests were incubated for 9 days. In the case of SRB presence, bacteria reduced sulfates present in kit to sulfides, which resulted in iron sulfide formation. The amount of precipitate was classified according to the kit manual. Each kit was certified by Droycon Bioconcepts Inc., Canada. Quality control was maintained by a parallel analysis of three blank samples for each series of measurements.

Total mercury concentration determination was performed via sediment sample (500 mg) pyrolysis in a stream of oxygen (Leco AMA 254, Czech Republic). The AMA254 technique of direct combustion features a combustion/catalyst tube where sediment decomposes in an oxygen-rich environment and is deprived of interfering elements. Both recovery and precision proved satisfactory (97 ± 3 % RSD, *n* = 5) based on reference material analysis (NIST 2584).

Methylmercury (MeHg) content was determined in the Josef Stefan Institute Laboratories in Ljubljana (Slovenia), using the procedure developed by Liang et al. (1994) and used successfully by others (Logar et al. 2002; Bełdowski et al. 2014; Quevauviller et al. 1998). All samples were analyzed in triplicate, and blank samples were run for every six samples. Recovery and precision of measurements were assessed by the use of certified reference material (BCR 580). Recovery equaled 91 % while RSDs did not exceed 7.4 % (*n* = 3).

Solid speciation of mercury was assessed by a sequential extraction procedure developed by Wallschlager et al. (1998) and adapted for marine sediments by Bełdowski and Pempkowiak (2003, 2007). Briefly, subsamples of wet sediments (containing some 2000 mg dry matter) were placed in polytetrafluoroethylene (PTFE) tubes and extracted overnight with 20 ml of a suitable extractant to obtain a given mercury fraction (Table 1). The extracts were used for Hg determinations, while the solid residue was subjected to the subsequent extraction. After the final extraction, the solid residue was acid digested in high pressure PTFE vessels. The assignment of mercury fractions is provided in Table 1; a detailed description and references to model compounds can be found in Wallschlager et al. (1998) and Bełdowski and Pempkowiak (2003).

**Table 1:** Procedure used for solid speciation of sedimentary mercury in marine sedimets

Fraction	Extractant	Extract treatment before CV-AAS measurement
Hg_A_	0.01 M HNO_3_	BrCl digestion (1 ml) followed by NH_2_OH·HCl (1 ml of 20 % solution). Hg dissolved and loosely adsorbed on sediment matrix
Hg_F,H_	1 M KOH	BrCl digestion (1 ml) followed by NH_2_OH·HCl (1 ml of 20 % solution) Hg bound to fulvic acids-HgF
		Precipitation at pH 2 and HNO_3_ hot digestion—Hg bound to humic acids-HgH
Hg_R_	HNO_3_/HClO_4_/HF	Oxidative digestion (120 °C/2 h in a teflon bomb, HNO_3_:HclO_4_ mixture 1.5:3 ml)—Hg incorporated in clay minerals lattice and bound to unextracted organic matter

Fraction description: *Hg*_*A*_ dissolved, *Hg*_*F,H*_ mercury bound to humic substances, *Hg*_*R*_ residual mercury

The solutions obtained in the course of sequential extraction were analyzed for mercury(II) content via the CV-AFS method in a Tekran 2600 model spectrophotometer (Canada). The analyses of the reference material QTMO56MS (from the QUASIMEME program) demonstrated that both accuracy and precision were satisfactory (recovery 92.5 ± 10 % RSD, *n* = 5). All results are provided on the dry and carbonate-free basis.

## Results and discussion

### Relation between methylmercury concentrations and sediment characteristics

Basic environmental characteristics influencing mercury methylation are well known (see “Introduction”). These include labile organic matter, labile mercury(II), total sulfur concentration, SRB abundance, and redox potential (Eh)—indicating reducing or at least anoxic conditions. Since the role of sulfur is the creation of stable mercury species, as cinnabar and mercury bound to other metal sulfides and fuelling sulfur reduction in sediments, it is represented here as resulting products. Mercury sulfidic fraction was extracted separately, and SRB abundance was measured directly. In order to get an indication of the strength of the properties’ relation to the methylation process in the investigated sedimentary environments, the relation of the individual measured sediment properties to MeHg concentration was assessed.

Linear dependencies were used to analyze existing relations, while determination coefficients were used as a measure of the relationship strength. Due to a large variability of the results, dependencies are presented per region (Table 2).

**Table 2:** Correlation coefficients of dependencies between the studied parameters and MeHg concentration in sediments

Region	No. of samples	Sediment properties studied						
		HgA	HgH	HgF	HgMob	POC	SRB	Eh
All*	**42**	**0.51**	**0.61**	**0.62**	**0.67**	**0.80**	**0.38**	−**0.49**
Balt Deep	24	−0.30	**0.80**	**0.75**	**0.88**	−0.03	**0.80**	−**0.49**
Bornholm Deep	6	−0.51	0.17	0.30	0.36	0.73	−0.39	−0.74
Gdańsk Deep	9	0.59	**0.78**	0.58	**0.97**	**0.95**	**0.76**	−**0.99**
Gotland Deep	9	−0.07	0.48	−0.15	0.41	0.11	0.56	−**0.80**
Balt Coast	**12**	**0.91**	**0.96**	**0.90**	**0.98**	**0.94**	**0.82**	−**0.95**
Odra mouth	6	0.76	0.41	0.57	0.81	0.81	na	−0.60
Vistula Mouth	**6**	**0.87**	**0.95**	**0.87**	**1.00**	**0.92**	0.80	−**0.93**
Spitsbergen	6	−0.17	−0.81	0.10	−0.57	−0.57	**0.99**	−0.93

Statistically significant coefficients are indicated as bold

*HgA* pore water mercury, *HgH* mercury complexed by humic acids, *HgF* mercury complexed by fulvic acids, *HgMob* sum of mobile mercury species, *POC* sedimentary organic carbon, *SBR* sulfur-reducing bacteria, *Eh* oxidation-reduction potential, *na* not enough data

*All-all samples analyzed: Balt Deep—Baltic sediment accumulation areas, Bornholm Deep, Gdańsk Deep, Gotland Deep, Balt Coast—Coastal Baltic samples, Odra mouth area, Vistula mouth area, Spitsbergen fjords

In some areas, the dependences are quite weak (Bornholm, Gotland), while in other areas, the dependences are statistically significant for all tested sediment properties (Baltic Coast, Gotland, Vistula). Understandably, the statistical significance thresholds depend on the number of samples analyzed.

Mobile mercury species tend to correlate with methylmercury better, as a sum of mobile species (HgMob) than as individual forms (HgA, HgH, HgF). This may be caused by a high variability of labile mercury forms, as their transformations in surface sediments are relatively fast—mercury can change equilibria among physicochemical forms and thus gather predominantly as some of them in the course of early diagenesis (Bełdowski and Pempkowiak 2007). Therefore, the sum of mobile mercury fractions (HgMob) seems to be better suited to characterize methylation substratum, than more specific species.

According to literature, methylation should be highly dependent on organic carbon content, which, in turn, is tightly bound to redox conditions in sediments (Schartup et al. 2013). These are best quantified as redox potential Eh (Jackson 1998; Choi et al. 1994). Although a negative correlation of MeHg with Eh is evident in the analyzed sediments, correlations to organic carbon content do not follow a simple pattern. This may result from the differences in sediment composition. Although the Baltic sediment accumulation basins are covered by muddy sediments, their distances to the shore, and hence to sources of terrestrial organic matter differ greatly, this is especially visible in the region of Gotland Deep. Winogradow and Pempkowiak (2014) registered an increased contribution of labile, autochthonous organic matter there, which supports the thesis that it is the labile organic matter that enhances methylation, a feature well established and originating in research on sedimentary methylation (Merritt and Amirbahman 2009).

It follows from the determination coefficients that in individual areas, different factors correlate best with methylmercury concentrations. Organic carbon seems to be dominant in coastal areas and the Gdańsk Deep region, directly affected by the Vistula inflow—again, most likely due to substantial, amounting to 60 % of the total, contribution of labile organic matter there (Winogradow and Pempkowiak 2014). A similar complex relation between methylmercury contribution to total mercury, as well as methylation rate was reported by Schartup et al. (2014) for estuaries in Northeastern US. In this study, negative organic carbon/methylmercury correlation was observed in pristine Spitsbergen areas, which may result from limited mercury availability for methylation, while a positive correlation is observed in anthropogenically impacted Baltic coastal sites. This suggests that OM rich sites have higher methylation rates, a fact previously reported by others (Schartup et al. 2013). Redox conditions seem to correlate with MeHg equally well in all studied areas, with a possible exception of the Odra mouth. Sandy sediments in this area are characterized with oxidative conditions (30–40 mEV). Although in reducing conditions methylation may be inversely correlated to negative redox potential, it seems that in oxidative conditions, after exceeding a certain threshold Eh value, methylation is very slow and is not related to positive values of redox potential.

The differences between correlations in individual areas (Table 2) suggest limited applicability of this approach for larger areas. This may be attributed to either the complex interplay of the measured factors or the influence of some other environmental factors affecting the methylation process that may prove locally important.

Areas which seem to be very specific in terms of methylation are Spitsbergen fjords. This is manifested by a concentration of methylmercury almost independent of the sediment properties. This may point to long range transport as an important source of MeHg present in Spitsbergen sediments (Bełdowski et al. 2015). On the other hand, it has been proven that (AMAP 2011; Bełdowski et al. 2015) mercury in the fjord sediment region can originate from atmospheric deposition, glacier meltwater, and rock weathering—so it can be assumed that at least part of the methylmercury observed there could be methylated prior to delivery to bottom sediments.

Therefore, an attempt was made to construct an equation based on the studied parameters, which could more accurately define the correlation to MeHg in large areas, irrespective of the region.

### Development and verification of the equation for calculating methylation index

The original methylation index previously developed by us (Bełdowski et al. 2009) had to be modified to reflect observed correlations and to maintain the biogeochemical relevance of the index. The original MI was calculated as a simple multiplication of the mercury mobile species concentration and loss on ignition, used as a measure of organic matter. As the redox was not included, such index had to be regarded separately for oxic and anoxic sediments, based on visual sediment sample classification.

**Fig. 2:**
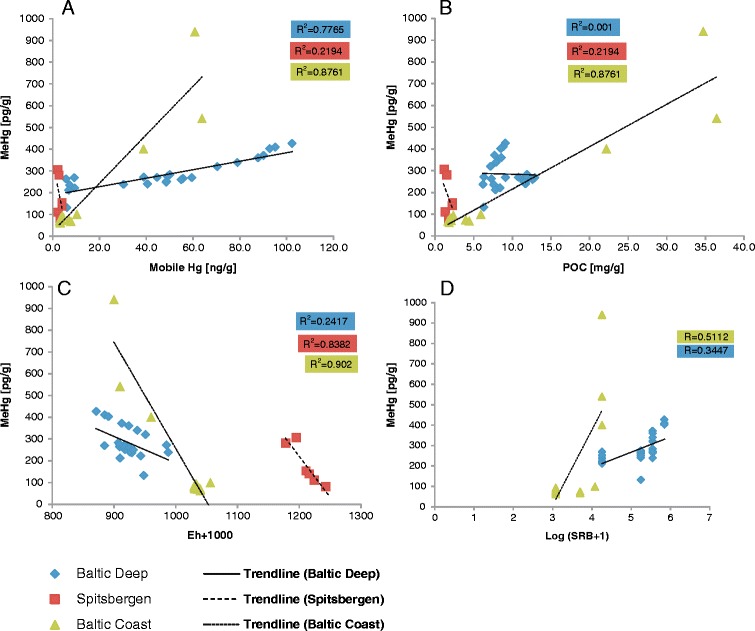
Scatterplots of parameters conditioning mercury methylation (**a** sum of mobile Hg, **b** organic carbon, **c** 1000+ Eh, and **d** log (SRB+1) versus methylmercury concentration [pg/g]

In order to include redox potential and sulfur-reducing bacteria abundance into the equation for MI calculation, both Eh and SRB values obtained from measurements were transformed. The following steps were taken: Eh was substituted with 1000+ Eh (mV)—in order to avoid negative numbers; while SRB values were log normalized (log (SRB+1)) to avoid large numbers and dynamics of the data. Unity in the SBR transformation was added to the equation in order to produce a value of 0 in cases when SRB were not detected. The linear dependences between methylmercury and the individual parameters were then tested. Analysis of scatterplots showed that linear correlation of the studied parameters exists, although it is evident that the slope of regression varies depending on the region (Fig. 2).

Since the studied parameters are well correlated to MeHg, it may be assumed that they contribute to methylation. Thus, a new equation that includes the properties of the sedimentary environment is required to replace the simple equation we proposed initially (Bełdowski et al. 2009). The sum of mobile species, as a substratum for methylation has to be included directly, while organic carbon and redox potential have to be connected to SRB, since in the absence of bacteria, methylation would be carried out along the abiotic pathway, which is considerably slower than the biotic one (Cossa et al. 2009; Krishnamurthy 1992). Since the abundance of methylmercury is directly proportional to organic carbon (POC) concentration and inversely proportional to the redox value, relation of POC to redox was chosen as representative, with the redox value incremented by 1000, to avoid negative numbers. The exponential relation of log normalized SRB to MeHg concentration was incorporated by choosing the exponent of this expression in the final MI equation. The new equation for calculating MI, including the properties most relevant for methylation, is proposed to be as follows:

$$ MI=\Sigma H{g}_{mob}\ast \left(\frac{POC}{1000+Eh}\right)\ast \exp \left( \log \left(SRB+1\right)\right) $$

**Fig. 3:**
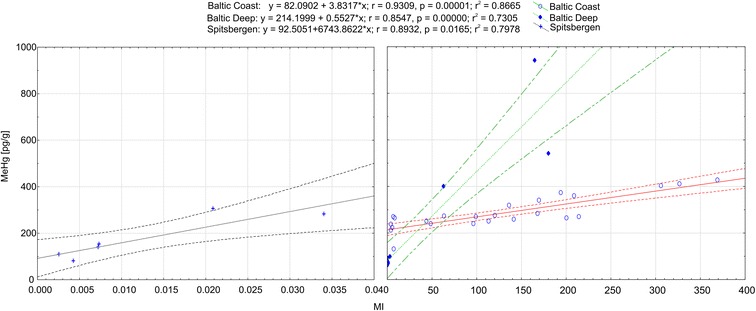
Relationship between methylation index and MeHg concentration [pg/g]. For clarity, MI values lower than 0.04 were presented in different scale

The generalization of the expression and lack of coefficients in this equation is deliberate. The authors decided that although the inclusion of purely mathematical constants could lead to a better reproduction of actual MeHg concentrations, the aim of MI should be the estimation of methylation capability, rather than modeling MeHg concentrations. Numerical values of the methylation index were calculated for all sediment samples used in the study. The calculated methylation index values were plotted against sedimentary methylmercury concentrations separately for all study areas (Fig. 3).

It seems that the overall correlation coefficients are slightly smaller than in the case of simple linear equations between MeHg and specific properties. Since the MI was developed to represent a potential of a given sedimentary environment to methylate labile mercury rather than the actual model aiming at reproducing MeHg concentration, some generalization was unavoidable. The best fit was achieved in Baltic coastal sediments, followed by Spitsbergen fjords, and sediments from the Baltic deeps; however, the degree of confidence is much smaller in Spitsbergen, where the methylation index range is also the smallest and much less variable. In the instance of the Baltic Deeps, the linear correlation coefficient is the smallest (0.73). This may be the consequence of the most numerous dataset, reflected in the best confidence level. Furthermore, elimination of one outlier at Bornholm Deep (MI = 7.54; MeHg = 131.6 pg/g) raises the correlation coefficient value to 0.75. Moreover, the Baltic Deep dataset comprises of samples originating from different environments (Winogradow and Pempkowiak 2014). A comparison between different datasets suggests that it is possible that the slope of linear relationship between MI and MeHg depends on the methylmercury source. In the Spitsbergen fjords, where little mobile mercury is present in the sediments, methylation most likely occurs either in the terrestrial environment or in the water column, where MeHg is produced and is subsequently deposited to marine sediments. In contrast, both methylated and inorganic mercury in coastal waters of the Baltic Sea are supplied by rivers (Saniewska et al. 2014). In the Baltic Deeps, where conditions within sediments favor in situ methylation, the slope of correlation is the smallest. It seems that this property of the methylation index could be used not only to predict methylation rates but also to indicate areas where methylmercury is transported from external sources. For comparison, the Michaelis-Menten equation proposed by Cossa et al. (2014) depicting MeHg relation to total mercury was applied to the dataset.

$$ MeHg=\frac{a\ast H{g}_T}{K_m+H{g}_T} $$

where MeHg represents predicted methylmercury concentration in nanomoles, Hg_T_ is nanomolar concentration of methylmercury, while empirically derived constants represent saturation MeHg concentration (a) (0.277 nmol/g) and *K*_m_ Hg_T_ concentration corresponding to MeHg half-saturation (188 nmol/g). When applied to data in this study, the correlation coefficients were comparable to these of the methylation index (Table 3), for Baltic sediments, showing even greater correlation. This may be the result of the application of empirical constants, whereas the methylation index is lacking any kind of calibration. This makes it a more universal indicator of methylation, as demonstrated in the pristine Spitsbergen area, where correlation level of the Michaelis-Menten equation is more than three times lower compared to the methylation index. This is most probably caused by a very low proportion of bioavailable mercury in those sediments—a fact included in MI but not in parametrical total mercury approach.

**Table 3:** Correlation coefficients and significance levels of methylation index and Michaelis-Menten equation to methylmercury concentration in studied sediments

	Methylation index		Michaelis-Menten	
Region	*R* ^2^	*p*	*R* ^2^	*p*
Baltic Deep	0.7305	0.00000	0.7765	0.00000
Baltic Coast	0.8665	0.00001	0.876	0.00001
Spitsbergen	0.7978	0.0165	0.219	0.3492

### Methylation index in study areas

**Fig. 4:**
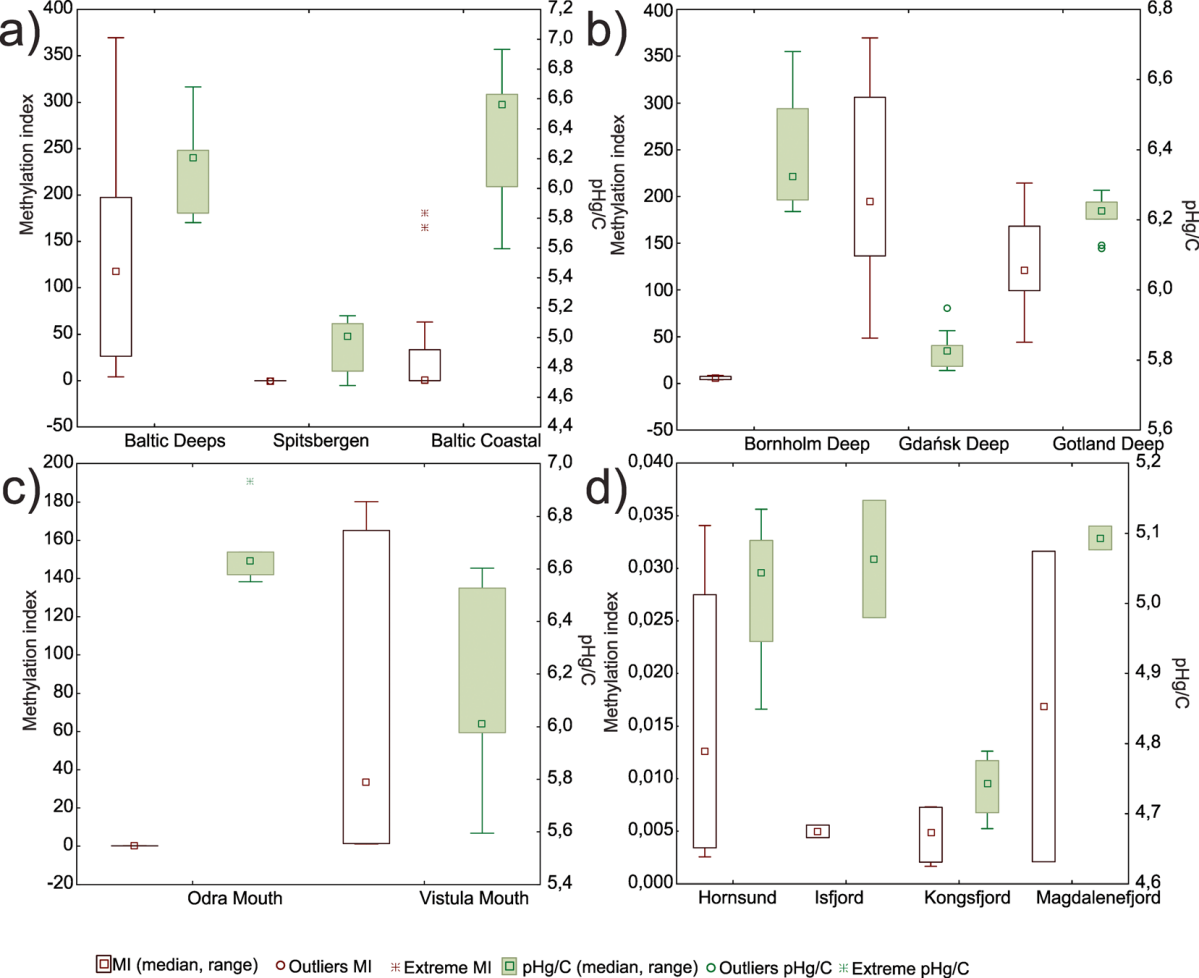
Methylation index and carbon-normalized mercury median values and ranges in particular study areas

Median values and ranges of the MI in the study subareas are shown in Fig. 4. In order to show an indicator of the anthropogenic pollution of those sediments, mercury normalized to carbon content (pHg/C = −log [Hg/C]) as proposed by Schartup et al. (2013) was used. Normality tests of MI dataset showed that the distribution is not normal (chi-squared test); therefore, Kruskal-Wallis ANOVA was used to assess the significance of the differences. Overall, the highest variability and the highest methylation indexes were observed in the Baltic Deeps and the smallest near Spitsbergen, while sediments of the Baltic Coastal areas were slightly more variable than those of Spitsbergen, with several outliers (Fig. 4a). The observed differences were significant (*p* = 0.000). The differences, of course, are caused by the variability of the factors used in the MI equation. One factor is especially variable as it characterizes both the anoxic environment (negative values of Eh) and the oxic environment (positive values of Eh). Therefore, the differences may be attributed to differences of the redox status of the sediments in question: Sediments of the Baltic Deeps represent anoxic environments with an abundant supply of mobile mercury and organic matter and are thus characterized by favorable conditions for mercury methylation, while coastal sediments in the southern Baltic Sea are usually oxic. Hence, favorable conditions for mercury methylation are achieved only during high supply of organic matter, e.g., due to algal blooms and/or the discharge of organic matter by rivers, followed by mineralization and local anoxia in sediments—explaining a relatively high MI of outliers. The Spitsbergen region, on the other hand, on top of oxic conditions in the sediments, is characterized by low values of mobile mercury, resulting in low in situ methylation (as deduced from correlations of MeHg and environmental factors). Mercury normalized to carbon varied from 4.6 in Spitsbergen to 6.9 in Baltic Coastal waters. Since mercury is assumed to follow organic carbon concentrations, lower pHg/C is often interpreted as excess mercury coming from anthropogenic pollution (Schartup et al. 2013). While this may be true for Baltic (deep basins showing overall higher human impact, due to the accumulation of fine sediments with elevated mercury concentration and respectively higher methylation index), it seems that in the case of pristine Spitsbergen areas, entirely different sedimentary environment causes different equilibria between organic carbon and mercury, making it impossible to compare to the Baltic.

Detailed examination of individual areas seems to confirm the environmental significance of the methylation index. In the Baltic, both the Gdańsk Deep and the Gotland Deep MI values are significantly different (*p* = 0.0009) from the Bornholm Deep ones—a fact likely explained by better oxygen conditions there (Fig. 4b). Bornholm Deep is located on the way of saline water inflows from the North Sea (Meier et al. 2006), which is well oxygenated, and can lead to the oxidation of the upper sediment layers, at least temporarily, hence inhibiting the methylation. Slightly higher methylation index in the Gdańsk Deep might be caused by larger organic matter supply and anthropogenic impact from the Vistula River, which results in a high percentage of labile mercury forms there (Bełdowski et al. 2009; Bełdowski et al. 2014). This is also confirmed by pHg/C values, showing the lowest values, corresponding to greater human impact in the Gdańsk Deep, medium values at Gotland, and the highest at Bornholm Deep.

MI values of coastal sediments in the Baltic (Fig. 4c) are also characterized by significant differences (*p* = 0.0039). Sediments of the Odra River mouth represent both a lower median and lower variability than the Vistula mouth sediments. This reflects the differences in the geographical settings of both areas—the Odra river is separated from the Baltic by the Szczecin Lagoon, which acts as a trap for organic matter (Pempkowiak et al. 2005), and mercury adsorbed to fine suspension, while Vistula discharges directly to the Gdańsk Bay. This reduces the variability of riverine anthropogenic material entering the mouth region and also decreases organic carbon content in the sediments of the Odra river mouth compared to the Vistula river mouth. Variable pHg/C values in the Vistula mouth correspond well with equally variable MI values observed there.

The methylation index in Spitsbergen fjords did not differ significantly (*p* = 0.816), although higher values could be observed in the Hornsund and Magdalene fjords. Those fjords are wide, and they are under the influence of Atlantic water moved by a western Spitsbergen current, which is supposed to bring more organic matter and heavy metals than water originating from rivers and glacier melting (Lu et al. 2013). It is remarkable, however, that MI values calculated for Spitsbergen are exceptionally low in comparison to the Baltic, reflecting unfavorable conditions for methylation there. Additionally, pHg/C values do not differ considerably, except for the Kongsfjord, and are generally lower than those observed in the Baltic. This is hardly associated with human impact, as mercury concentrations in the Kongsfjord are not elevated, and no local pollution was identified there (Bełdowski et al. 2015). Since no expected inverse correlation is observed with the methylation index, it seems that the dependencies between organic matter and mercury in pristine areas, where mercury deposition is episodic, and carbon sources are associated with primary production rather than with terrestrial inputs, opposite to those observed in anthropogenically impacted areas. This further substantiates the use of more sophisticated indexes, based on more variables for inter area comparison.

## Conclusions

The methylation index has been developed as a characteristic of surface sediments indicating potential for sedimentary mercury to be methylated and to persist as methylmercury. In order to control the consistency of MI with MeHg abundance, several sedimentary environments differing with organic matter and sedimentary mercury concentrations were investigated. The obtained index has been validated against methylmercury concentration in sediments, to obtain satisfactory agreement (range of *R*^2^ = 0.73 to 0.89, depending on region). Analysis of the methylation index in particular regions show that MI values reflect environmental conditions required for mercury to be methylated in those areas. However, the calculated values do not directly reproduce MeHg concentrations. This leads to the conclusion that MI could be used in methylation rate modeling, provided that empirical constants coming from radiotracer studies could be applied to improve MeHg reproducibility. MI could be also used as general characteristic of the sedimentary environment with respect to MeHg occurrence.

## References

[CR1] AMAP (2011). *AMAP assessment 2011: mercury in the Arctic*. Oslo.

[CR2] Avramescu ML, Yumvihoze E, Hintelmann H, Ridal J, Fortin D, Lean DRS (2011). Biogeochemical factors influencing net mercury methylation in contaminated freshwater sediments from the St. Lawrence River in Cornwall, Ontario, Canada. Science of the Total Environment.

[CR3] Bełdowski J, Pempkowiak J (2003). Horizontal and vertical variabilities of mercury concentration and speciation in sediments of the Gdansk Basin, Southern Baltic Sea. Chemosphere.

[CR4] Bełdowski J, Pempkowiak J (2007). Mercury transformations in marine coastal sediments as derived from mercury concentration and speciation changes along source/sink transport pathway (Southern Baltic). Estuarine, Coastal and Shelf Science.

[CR5] Bełdowski J, Miotk M, Pempkowiak J (2009). Mercury fluxes through the sediment water interface and bioavailability of mercury in southern Baltic Sea sediments. Oceanologia.

[CR6] Bełdowski J, Miotk M, Bełdowska M, Pempkowiak J (2014). Total, methyl and organic mercury in sediments of the Southern Baltic Sea. Marine Pollution Bulletin.

[CR7] Bełdowski J, Miotk M, Zaborska A, Pempkowiak J (2015). Distribution of sedimentary mercury off Svalbard, European Arctic. Chemosphere.

[CR8] Boening DW (2000). Ecological effects, transport, and fate of mercury: a general review. Chemosphere.

[CR9] Borg H, Jonsson P (1996). Large-scale metal distribution in Baltic Sea sediments. Marine Pollution Bulletin.

[CR10] Boszke, L., Kowalski, A., & Siepak, J. (2007). Fractionation of mercury in sediments of the Warta River (Poland). In Pawłowski, Dudzińska & Pawłowski (Eds), *Environmental Engineering* (pp. 403–413). London: Taylor and Francis Group.

[CR11] Buccolieri A, Buccolieri G, Cardellicchio N, Dell'Atti A, Di Leo A, Maci A (2006). Heavy metals in marine sediments of Taranto Gulf (Ionian Sea, Southern Italy). Marine Chemistry.

[CR12] Choi SC, Chase T, Bartha R (1994). Metabolic pathways leading to mercury methylation in desulfovibrio-desulfuricans LS. Applied and Environmental Microbiology.

[CR13] Cossa D, Averty B, Pirrone N (2009). The origin of methylmercury in open Mediterranean waters. Limnology and Oceanography.

[CR14] Cossa D, Garnier C, Buscail R, Elbaz-Poulichet F, Mikac N, Patel-Sorrentino N (2014). A Michaelis-Menten type equation for describing methylmercury dependence on inorganic mercury in aquatic sediments. Biogeochemistry.

[CR15] Figueiredo N, Serralheiro ML, Lino AR, Carvalho C (2011). Biochemical characterization of sulphate reducing bacteria isolated from Tagus Estuary (Lisbon, Portugal) exhibiting resistance to mercury compounds to evaluate their contribution to mercury cycle. Toxicology Letters.

[CR16] Harmon SM, King JK, Gladden JB, Newman LA (2007). Using sulfate-amended sediment slurry batch reactors to evaluate mercury methylation. Archives of Environmental Contamination and Toxicology.

[CR17] Heyes A, Mason RP, Kim EH, Sunderland E (2006). Mercury methylation in estuaries: insights from using measuring rates using stable mercury isotopes. Marine Chemistry.

[CR18] Hintelmann H, Ogrinc N (2003). Determination of stable mercury isotopes by ICP/MS and their application in environmental studies. Biogeochemistry of Environmentally Important Trace Elements.

[CR19] Hintelmann H, Keppel-Jones K, Evans RD (2000). Constants of mercury methylation and demethylation rates in sediments and comparison of tracer and ambient mercury availability. Environmental Toxicology and Chemistry.

[CR20] Jackson TA, Langston J, Bebiano MJ (1998). Mercury in aquatic ecosystem. Metal metabolism in aquatic environment.

[CR21] Krishnamurthy S (1992). Biomethylation and environmental transport of metals. Journal of Chemical Education.

[CR22] Liang L, Horvat M, Bloom NS (1994). An improved speciation method for mercury by Gc Cvafs after aqueous-phase ethylation and room-temperature precollection. Talanta.

[CR23] Logar M, Horvat M, Akagi H, Pihlar B (2002). Simultaneous determination of inorganic mercury and methylmercury compounds in natural waters. Analytical and Bioanalytical Chemistry.

[CR24] Lu ZB, Cai MH, Wang J, Yin ZG, Yang HZ (2013). Levels and distribution of trace metals in surface sediments from Kongsfjorden, Svalbard, Norwegian Arctic. Environmental Geochemistry and Health.

[CR25] Martinez SS, Gallegos AA, Martinez E (2004). Electrolytically generated silver and copper ions to treat cooling water: an environmentally friendly novel alternative. International Journal of Hydrogen Energy.

[CR26] Meier HEM, Feistel R, Piechura J, Arneborg L, Burchard H, Fiekas V (2006). Ventilation of the Baltic Sea deep water: a brief review of present knowledge from observations and models. Oceanologia.

[CR27] Merritt KA, Amirbahman A (2008). Methylmercury cycling in estuarine sediment pore waters (Penobscot River estuary, Maine, USA). Limnology and Oceanography.

[CR28] Merritt KA, Amirbahman A (2009). Mercury methylation dynamics in estuarine and coastal marine environments—a critical review. Earth-Science Reviews.

[CR29] Monperrus M, Tessier E, Amouroux D, Leynaert A, Huonnic P, Donard OFX (2007). Mercury methylation, demethylation and reduction rates in coastal and marine surface waters of the Mediterranean Sea. Marine Chemistry.

[CR30] Pak KR, Bartha R (1998). Mercury methylation and demethylation in anoxic lake sediments and by strictly anaerobic bacteria. Applied and Environmental Microbiology.

[CR31] Pempkowiak J (1991). Enrichment factors of heavy-metals in the Southern Baltic surface sediments dated with Pb-210 and Cs-137. Environment International.

[CR32] Pempkowiak J, Cossa D, Sikora A, Sanjuan J (1998). Mercury in water and sediments of the southern Baltic Sea. Science of the Total Environment.

[CR33] Pempkowiak J, Beldowski J, Pazdro K, Staniszewski A, Zaborska A, Leipe T (2005). Factors influencing fluffy layer suspended matter (FLSM) properties in the Odra River—Pomeranian Bay—Arkona Deep System (Baltic Sea) as derived by principal components analysis (PCA), and cluster analysis (CA). Hydrology and Earth System Sciences.

[CR34] Quevauviller P, Andersen K, Merry J, van der Jagt H (1998). Interlaboratory study to improve the quality of trace element determinations in groundwater. Analyst.

[CR35] Richir J, Velimirov B, Poulicek M, Gobert S (2012). Use of semi-quantitative kit methods to study the heterotrophic bacterial community of Posidonia oceanica meadows: limits and possible applications. Estuarine, Coastal and Shelf Science.

[CR36] Saniewska D, Beidowska M, Beldowski J, Jedruch A, Saniewski M, Falkowska L (2014). Mercury loads into the sea associated with extreme flood. Environmental Pollution.

[CR37] Schartup AT, Mason RP, Balcom PH, Hollweg TA, Chen CY (2013). Methylmercury production in estuarine sediments: role of organic matter. Environmental Science & Technology.

[CR38] Schartup AT, Balcom PH, Mason RP (2014). Sediment-porewater partitioning, total sulfur, and methylmercury production in estuaries. Environmental Science & Technology.

[CR39] Wallschlager D, Desai MVM, Spengler M, Windmoller CC, Wilken RD (1998). How humic substances dominate mercury geochemistry in contaminated floodplain soils and sediments. Journal of Environmental Quality.

[CR40] Winogradow A, Pempkowiak J (2014). Organic carbon burial rates in the Baltic Sea sediments. Estuarine, Coastal and Shelf Science.

